# LncRNA LINC00152 Promotes Laryngeal Cancer Progression by Sponging MiR-613

**DOI:** 10.1515/med-2020-0035

**Published:** 2020-03-26

**Authors:** Xuesong Zheng, Su Dong, Lele Sun, Jialu Xu, Jia Liu, Rui Hao

**Affiliations:** 1 Department of Infection, The Affiliated Hospital of Beihua University, Jilin 132001, P.R. China; 2 Department of Otolaryngology Head and Neck surgery, the Affiliated Hospital of Beihua University, Jilin 132001, P.R. China; 3 Departments of Anesthesia, the First Hospital of Jilin University, Changchun 130021, P.R. China; 4 Department of Thyroid Surgery, The First Hospital of Jilin University, Changchun 130021, P.R. China

**Keywords:** LINC00152, long noncoding RNA, laryngeal squamous cell cancer, miR-613

## Abstract

**Background:**

Long noncoding RNA (lncRNA) LINC00152 (CYTOR) has been reported to be upregulated and to serve as a diagnostic biomarker in multiple types of cancers, including laryngeal squamous cell cancer (LSCC). However, the functional role and molecular mechanisms of LINC00152 in LSCC progression need to be further investigated.

**Methods:**

LINC00152 levels in LSCC and adjacent normal tissues were measured by quantitative real-time polymerase chain reaction (qRT-PCR). Gene knockdown of LINC00152 was achieved in LSCC cells by use of small interfering RNA (siRNA). Cell proliferation, apoptosis, migration and invasion were examined by a series of methods. The micoRNA (miRNA) interaction with LINC00152 was screened by starBase v2.0 and confirmed by luciferase reporter activity.

**Results:**

LINC00152 levels in LSCC tissues were significantly higher than those in adjacent normal tissue, and patients with lymph node metastasis or an advanced clinical stage displayed higher LINC00152 expression. Moreover, siRNA-mediated LINC00152 knockdown significantly inhibited the proliferation, migration and invasion of LSCC cells and induced apoptosis in those cells. Mechanistically, LINC00152 functioned as a competing endogenous RNA (ceRNA) sponging miR-613. The inhibitory effect of LINC00152 knockdown on malignant behavior was abrogated by inhibiting miR-613.

**Conclusion:**

LINC00152 exerts an oncogenic effect on the tumorigenesis of LSCC by sponging miR-613 and may serve as a potential target for treating LSCC.

## 1 Introduction

Laryngeal squamous cell carcinoma (LSCC) is the most common malignancy of the upper respiratory tract and is the second most common cancer among head and neck malignancies [1]. Although much progress has been made in treatment, including surgical intervention, radiation therapy and chemotherapy, the prognosis for patients with advanced LSCC remains unsatisfactory [2,3]. Thus, it is crucial to explore the molecular mechanisms underlying LSCC carcinogenesis or progression to develop a more effective therapy target for this disease.

Long non-coding RNA(lncRNA), an integral part of the human genome, is longer than 200 nucleotides, with limited protein-coding ability [4]. LncRNAs have been reported to play crucial roles in diverse cellular processes, including proliferation, apoptosis, differentiation and metastasis [5,6]. Moreover, lncRNAs identified in the initiation and development of tumors function as oncogenes and tumor suppressors [7,8]. Accumulating evidence indicates that lncRNA is implicated in tumor progression through its interactions with other cellular macromolecules, including RNA, DNA, and protein [9,10]. A number of studies have demonstrated that lncRNA has the potential as a biomarker or therapy target for LSCC [11,12]. Therefore, there is urgent need to explore the role of lncRNA in LSCC progression.

Long intergenic noncoding RNA 152 (LINC00152), located at chromosome 2p11.2[13], has been documented as an oncogene in multiple types of cancer, including colorectal[14] gastric[15], breast[16], glioma [17], lung[18] hepatocellular[19] and renal cell [20]. A recent study has revealed that LINC00152 is highly expressed in LSCC tissue [21]. However, the exact biological role and potential mechanism of LINC00152 in LSCC have not been well known until now. Therefore, the aims of this study were to investigate the role of LINC00152 in LSCC and the potential mechanism *in vitro*. We found that LINC00152 is upregulated in human LSCC. Moreover, LINC00152 accelerated the malignant progression of LSCC cells through sponging miR-613, revealing a novel mechanism underlying the contribution of LINC00152 to LSCC development.

## 2 Materials and method

### 2.1 Patients and tissue specimens

Forty pairs of LSCC tissue samples and corresponding adjacent non-cancerous tissue were harvested from patients with LSCC at the Department of Otolaryngology, Head and Neck Surgery, the Affiliated Hospital of Beihua University (Jilin, China) from January, 2017 to January, 2018. The adjacent normal tissues were collected from the resected tissue of 40 patients with LSCC, approximately 1 cm away from the tumors. The diagnosis was obtained by pathological examination. Informed consent was gained from all patients or family; no patients had received chemo-radiotherapy or other therapy before surgery. The use of human tissue specimens was approved by the Ethics Committee of the Beihua University. All specimens were placed into RNA later solution (CoWin Biosciences, Beijing, China) and stored at -80˚C until RNA extraction. The clinicopathologic characteristics of the 40 patients are presented in Table 1.

**Table 1:** Association of LINC00152 expression with clinicopathologic factors in 40 cases of patients with LSCC.

Variables	No. of cases	LINC00152 expression		*p* value^^a^^
		High	Low	
Age(years)				*p*=0.5254
<60	18	8	10	
≥60	22	13	9	
Sex				*p*=0.1965
Male	24	15	9	
Female	16	6	10	
UICC stage				***P*=0.0214**
I-II	31	13	18	
III-IV	9	8	1	
Histological differentiation				*p*=0.2812
Well and moderately	30	14	16	
Poor	10	7	3	
Lymph node metastasis				***P*<0.0001**
No	32	13	19	
Yes	8	8	0	

a Statistical significant results (in bold)

### 2.2 Cell culture and transfection

Three LSCC cell lines (SCC-2, SCC-40 and TU212) and a normal bronchial epithelial cell line (16HBE) were purchased from the Chinese Academy of Science of Shanghai (Shanghai, China), and grown in RPMI-1640 (Gibco, CA, USA) with 10% fetal bovine serum (FBS, Gibco) under a humidified incubator with 5% carbon dioxide at 37°C.

Three specific small interfering RNA (siRNA) oligo targeting LINC00152 in different regions were mixed and named si-LINC00152. The control siRNA (si-NC) and si-LINC00152 were purchased from GenePharma (Shanghai, China). The mimics and inhibitor of miR-613, as well as their negative control mimics or inhibitor (miR-NC or anti-miR-NC), were purchased from Ribobio Co. (Guangzhou, China). TU212 cells were plated in 6-well plates and grown to 70% confluence. Subsequently, the siRNA oligo, mimics or inhibitor were transfected into TU212 cells using Lipofectamine 2000 (Invitrogen, Carlsbad, CA, USA), according to the manufacturer’s instructions.

### 2.3 Real-Time Quantitative RT-PCR Analysis

Total RNA was extracted from tissues and cell lines using Trizol reagent (Invitrogen), according to the manufacturer’s instructions. Reverse transcription was done with the iScript^TM^ cDNA Synthesis Kit (Bio-Rad, Hercules, CA, USA).Real-time PCR was conducted with a SYBR^®^Premix ExTaq™ (TAKARA BIO INC, Japan) Kit on ABI7500 Fast system. GAPDH and U6 were used as internal controls to detect LINC00152 or miR-613, respectively. The primes used in this study have been described previously [15,22]. Relative expression was calculated using the 2^−△△Ct^ method.

### 2.4 Proliferation Assay

Cell proliferation was examined with the Counting Kit 8 (CCK8) (Dojindo, Laboratories, Kumamoto, Japan). Transfected cells (5×103) were seeded into 96-well plates and cultured for 24, 48 and 72 hours (h). CCK8 solution was added to each well 4 h before the end of the culture time. Then, the absorbance of a well at 450 nm was measured with a microplate reader (Bio-Rad, Hercules, CA, USA).

### 2.5 Apoptosis Assay

The apoptosis assay was performed with a PE Annexin V Apoptosis Detection Kit (BD Biosciences, USA), according to the manufacturer’s instructions.

### 2.6 Wound healing assay

Transfected cells were placed into 6-well culture plates and grew to 100% confluence. The wound area was created by scratching the cell monolayer. After washing with PBS, the cells were cultured in free-serum medium for 24 h. Scratch wounds were imaged in the same position at 0 h and 24 h after wounding via an Olympus microscope (Tokyo, Japan).

### 2.7 Transwell assays

The Transwell chambers (8mm pore filter; Corning Company, New York, USA) coated with Matrigel (BD Biosciences, San Jose, CA, USA) were used to test cell invasion. Briefly, 48 h posttransfection, 200 μL cell suspension in serum-free medium was placed in the upper chamber, and 500 μL of complete RPM1640 medium containing 10% FBS was added to the lower chamber. After 24 h, invaded cells remaining in the lower chamber were fixed with methanol for 30 minutes and stained with 1% crystal violet solution for 1 h. The stained cells were imaged and counted in 5 randomly chosen fields with an inverted microscope (Olympus Corporation, Tokyo, Japan).

### 2.8 Luciferase report assay

The possible binding sites between LINC00152 and miR-613 were predicted with starBase v2.0. The LINC00152 fragments with the predicted wild type (WT) miR-613 binding sites were amplified and sub-cloned downstream of firefly luciferase report plasmid in pmirGLO reporter vector (Promega, Madison, WI, USA) and named as LINC00152-WT. The GeneTailor™ Site-Directed Mutagenesis System (Invitrogen, USA) was used to construct a mutant type of LINC00152 (LINC00152-MUT). The luciferase reporter plasmids (LINC00152-WT or LINC00152-MUT) were co-transfected with miR-613 mimics or miR-NC into TU212 cells. Forty-eight hours later, the luciferase assay was conducted with the dual-luciferase reporter assay system.

### 2.9 Statistical analysis

The analysis of statistical differences was performed with use of SPSS 20.0 (Chicago, IL, USA) with Student’s *t*-test (between 2 groups) or one-way ANOVA (three or more groups). All results are shown as means ± standard deviation (SD) from at least three independent experiments. The relationship between LINC00152 and clinicopathologic factors was analyzed by Pearson chi-square tests. Correlation between miR-613 expression and LINC00152 was analyzed by the Spearman’s test. Statistical significance is presented as *P* < 0.05.

## 3 Results

### 3.1 LINC00152 was significantly upregulated in LSCC tissues and associated with poor prognosis

**Figure 1:**
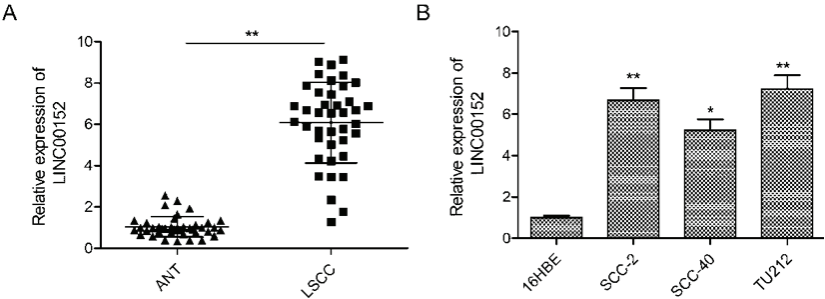
LINC00152 was significantly upregulated in LSCC tissue and cell lines. (A) The expression of LINC00152 was increased in LSCC tissue compared to adjacent normal tissue (ANT). (B) The expression of LINC00152 was increased in three LSCC cell lines (SCC-2, SCC-40 and TU212) compared to normal bronchial epithelial cell line ( 16HBE). **P < 0.01.

The expression of LINC00152 in LSCC was evaluated by qRT-PCR in 40 pairs of LSCC tissues and adjacent normal tissue. As shown in Figure 1A, the expression of LINC00152 was significantly upregulated in LSCC tissue compared with adjacent normal tissue (6.086 vs.1.043). Further investigation revealed that the expression of LINC00152 in LSCC cell lines (SCC-2, SCC-40 and TU212) was consistently higher than in normal cell lines (Figure 1B).

To explore the association between LINC00152 expression and clinicopathologic features, we divided enrolled patients into two groups based on LINC00152 expression value: LINC00152 high group (> mean; 21 cases) and LINC00152 low group (< mean; 19 cases). As shown in Table 1, the expression level of LINC00152 in LSCC tissue was significantly correlated with the clinical UICC (Union for International Cancer Control) stage (*p*=0.0214) and lymph node metastasis (*p*<0.0001). There was no correlation between LINC00152 level and age, gender or histologic differentiation (P>0.05). These results implied that LINC00152 promotes LSCC development.

### 3.2 LINC00152 depletion inhibits cell proliferation and induces apoptosis of LSCC cells

**Figure 2:**
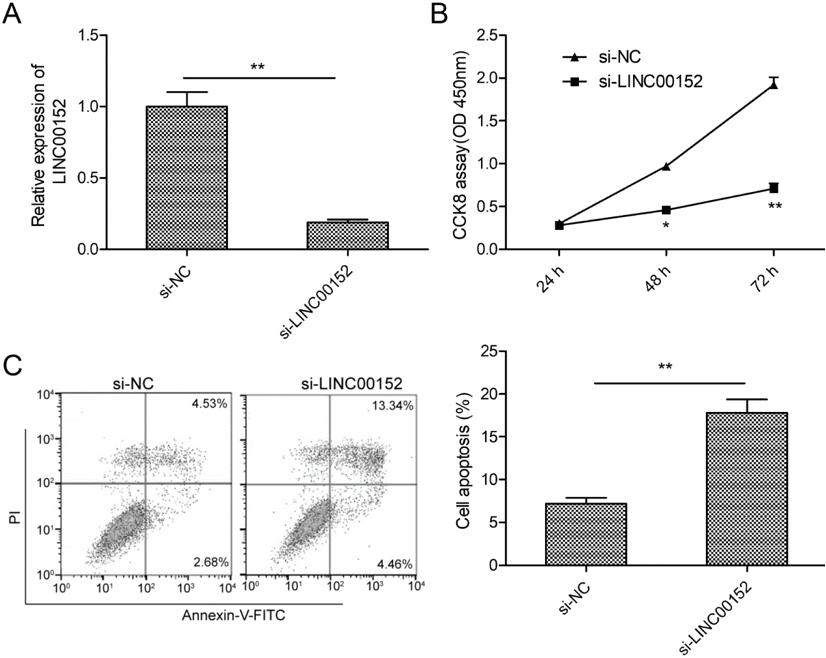
LINC00152 depletion inhibits cell proliferation and induces apoptosis of LSCC cells. (A) The transfected efficiency of si-NC and si-LINC00152 was confirmed in TU212 cells through qRT-PCR. (B, C) The proliferation and apoptosis of TU212 cells transfected with si-LINC00152 or si-NC were examined by CCK8 and flow cytometric assays, respectively. **P< 0.01.

To investigate the potential effect of LINC00152 in LSCC cells, we successfully knocked out LINC00152 in TU212 cells, which has a higher expression of LINC00152, by transfection with si-LINC00152 (Figure 2A). A CCK8 assay revealed that knockdown of LINC00152 significantly inhibited proliferation of TU212 cells at 48 h-72h (Figure 2B). Flow cytometric assay demonstrated that LINC00152 depletion obviously induced apoptosis of TU212 cells (Figure 2C).

### 3.3 LINC00152 depletion inhibits cell migration and invasion of LSCC cells

**Figure 3:**
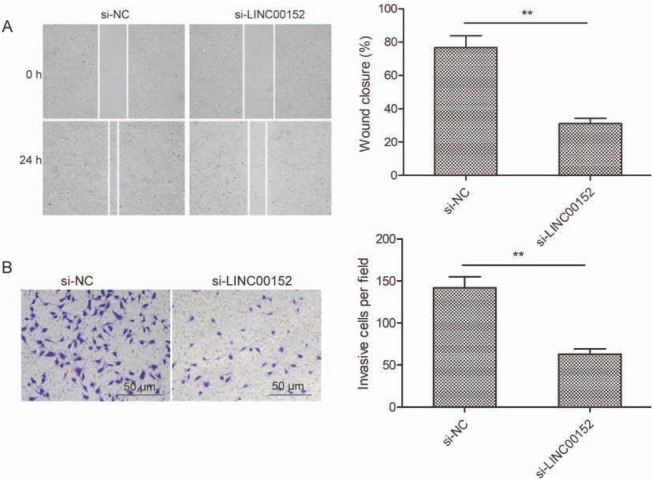
LINC00152 depletion inhibits invasion and migration of LSCC cells. (A) The migration ability was examined in TU212 cells transfected with si-LINC00152 or si-NC by wound healing assay.(B) The invasion ability was examined in TU212 cells transfected with si-LINC00152 or si-NC by Transwell invasion assay. **P < 0.01.

To detect whether LINC00152 could regulate LSCC metastasis, we determined migration and invasion of TU212 by performing wound healing and Transwell invasion assays, respectively. Wound healing assay revealed that LINC00152 depletion displayed a slower closing of a scratch wound compared with si-NC group in TU212 cells(-Figure3A). The Transwell assay revealed that the invasion cell numbers of TU212 cells transfected with si-LINC00152 were remarkably reduced compared with si-NC group (Figure 3B). These findings suggested that knockdown of LINC00152 hindered cell migration and invasion in TU212 cells.

### 3.4 LINC00152 regulates miR-613 expression by direct interaction in LSCC cells

**Figure 4:**
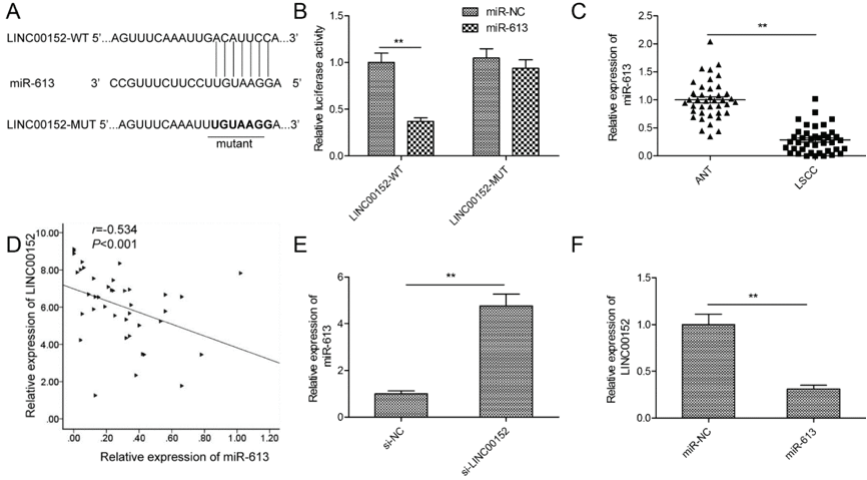
LINC00152 regulates miR-613 expression by direct interaction in LSCC cells. (A) The complementary binding of miR-613 and wild/ mutant type of LINC00152 is shown. WT: Wild-type; MUT: mutant-type. (B) Overexpression of miR-613 significantly reduced the luciferase activity of WT-LINC00152 group. (C) qRT-PCR analysis showed the expression of miR-613 in LSCC tissue and adjacent normal tissue (ANT). (D) The correlation between LINC00152 and miR-613 was analyzed in LSCC tissues by Spearman’s test. (E) The expression of miR-613 was examined in TU212 cells transfected with si-NC or si-LINC00152 by qRT-PCR. (F) The expression of LINC00152 was examined in TU212 cells transfected with miR-613 mimics or miR-NC by qRT-PCR. *P < 0.05; **P< 0.01.

Accumulating evidence supports lncRNA exerting a biological role by functioning as competing endogenous RNA (ceRNA) through competitively binding to miRNA to regulate miRNA expression [7]. To investigate the molecular mechanisms of LINC00152 in LSCC progression, the starBase v2.0 online website (http://starbase.sysu.edu.cn/mirLncRNA.php) was applied to predict potential miRNA that have a chance to interact with LINC00152. Among types of miRNA, miR-613 was selected as a study object based on its biological function [23]. As displayed in Figure 4A, complementary sites existed between LINC00152 and miR-613, hinting of the possible interaction of LINC00152 and miR-613. To further substantiate this prediction, a luciferase reporter activity assay was performed. It was found that overexpression of miR-613 caused a reduction of luciferase activity of LIN00152-WT in TU212 cells, but not that of LINC00152-MUT, suggesting LINC00152 could interact with miR-613 by putative binding sites in LSCC cells (Figure 4B). Subsequently, a qRT-PCR assay further revealed that the expression of miR-613 was remarkably downregulated in LSCC tissue (Figure 4C), and its expression was inversely correlated with LINC00152 level in 40 cases including LSCC tissues (Figure 4D). Additionally, knockdown of LINC00152 resulted in a noticeable increase of miR-613 level in TU212 cells (Figure 4E), while overexpression of miR-613 significantly inhibited LINC00152 expression in TU212 cells (Figure 4F). Taken together, these results suggested that LINC00152 functions as a ceRNA for sponging miR-613.

### 3.5 The miR-613 inhibition abrogated the inhibitory effects of LINC00152 depletion on cell proliferation, migration and invasion in LSCC

**Figure 5:**
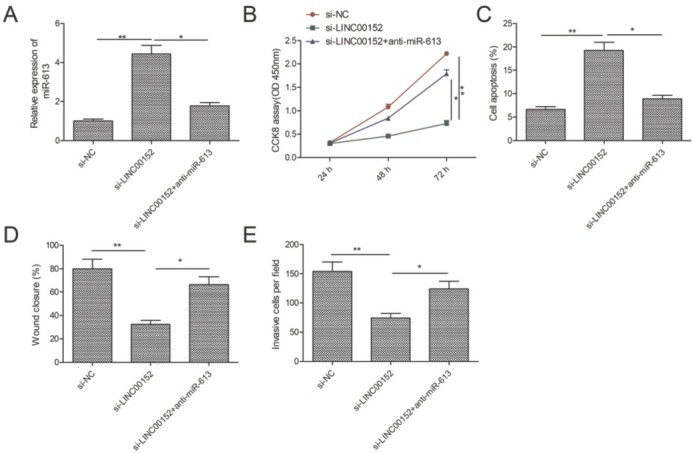
The miR-613 inhibition abrogated the effects of LINC00152 depletion on cell proliferation, migration and invasion in LSCC. (A) The expression of miR-613 was determined in TU212 cells transfected with si-NC, si-LINC00152, si-LINC00152+ miR-613 inhibitor (anti-miR-613) by qRT-PCR. (B-E) Cell proliferation, apoptosis, migration and invasion were determined in TU212 cells transfected with si-NC, si-LINC00152, si-LINC00152+ anti-miR-613. * P< 0.05; **P < 0.01.

To confirm whether the effect of LINC00152 on LSCC cells is dependent on miR-613, a rescue experiment was performed. We found that inhibition of miR-613 could partially rescue the upregulation of miR-613 caused by LINC00152 depletion in TU212 cells (Figure 5A). Moreover, the miR-613 inhibitor partially abrogated the effects of LINC00152 depletion on cell proliferation, apoptosis, migration and invasion in LSCC (Figure 5B-E), suggesting that LINC00152 promoted LSCC progression by regulating miR-613.

## 4 Discussion

The initiation and development of LSCC is a complicated process driven by the dysregulation of multiple genes [24]. Among these dysregulated genes, lncRNA has a crucial role in LSCC progression and functions as either tumor suppressor or oncogene [9,10]. For example, CAS5 functions as a tumor suppressor in LSCC progression through the negative regulation of miR-21[25]. SNHG12 promotes LSCC cell progression via regulating the miR-129-5p/WWP1 axis [26]. MIR100HG promotes LSCC growth and metastasis through the downregulation of miR-204-5p [27]. We found that LINC00152 was upregulated and functioned as an oncogene in LSCC by regulating miR-613, suggesting that LINC00152 might be a potential target for diagnosis and therapy.

Several studies have demonstrated that LINC00152 is upregulated and functions as a tumor promoter in many cancers [13, 14, 15, 16, 17, 18, 19, 20]. Although LINC00152 is reported to be upregulated in LSCC tissues [21], the exact biological role and potential mechanism of LINC00152 remain unclear. In the present study, we found that LINC00152 was abnormally upregulated in LSCC tissue and cells, which was consistent with previous reports [21]. Our results also revealed that LINC00152 depletion could repress the proliferation, induce apoptosis, and inhibit migration and invasion in LSCC cells. These results suggest that LINC00152 plays an oncogenic role in LSCC.

Accumulating evidence suggests that lncRNA exerts its biologic role by binding with miRNA as a sponge since it contains motifs with sequences complementary to miRNA [28]. LINC00152 has been reported to interact with multiple miRNAs including miR-376-3c[29], miR-497[30], miR-139-5p[31], miR-4775[17], miR-107[32], miR-193a-3p[15], miR-153-3p[33] and miR-125b[34]. Therefore, we hypothesized that LINC00152 might also be implicated in affecting the tumorigenesis and progression in LSCC through a similar mechanism. We predicated the potential miRNA targets of LINC00152 by using starBase v2.0 and found that miR-613 might be a potential target of LINC00152. Through a luciferase reporter activity assay, we confirmed that LINC00152 could interact with miR-613. MiR-613, a known tumor suppressor miRNA, has been reported to be downregulated in multiple cancers [22,35, 36, 37, 38]. In LSCC, miR-613 overexpression inhibits LSCC cell proliferation, invasion and blocks G1/S phase transition by targeting 3-phosphoinositide-dependent protein kinase-1 (PDK1) [39]. In our study, we found that miR-613 expression was downregualted in LSCC, which was consistent with previous studies[39]. In addition, miR-613 expression was negatively correlated with LINC00152 in LSCC tissues. Furthermore, miR-613 inhibition partially abrogated the effects of LINC00152 depletion on cell proliferation, apoptosis, migration and invasion in LSCC. These results suggest that LINC00152 promotes LSCC progression by sponging miR-613.

In summary, our study indicates that highly expressed LINC00152 is positively associated with advanced UICC stage and lymph node metastasis of LSCC. LINC00152 functions as an oncogenic lncRNA that contributes to tumorigenesis and progression of LSCC by sponging miR-613. These findings suggest that LINC00152 might be a new target for LSCC therapy.

## References

[1] Chu EA, Kim YJ (2008). Laryngeal cancer: diagnosis and preoperative work-up. Otolaryngologic clinics of North America.

[2] Ferlay J, Shin HR, Bray F, Forman D, Mathers C, Parkin DM (2010). Estimates of worldwide burden of cancer in 2008: GLOBOCAN 2008. International journal of cancer.

[3] Rudolph E, Dyckhoff G, Becher H, Dietz A, Ramroth H (2011). Effects of tumour stage, comorbidity and therapy on survival of laryngeal cancer patients: a systematic review and a meta-analysis. European archives of oto-rhino-laryngology : official journal of the European Federation of Oto-Rhino-Laryngological Societies.

[4] Kornienko AE, Guenzl PM, Barlow DP, Pauler FM (2013). Gene regulation by the act of long non-coding RNA transcription. BMC biology.

[5] Ponting CP, Oliver PL, Reik W (2009). Evolution and functions of long noncoding RNAs. Cell.

[6] Geisler S, Coller J (2013). RNA in unexpected places: long non-coding RNA functions in diverse cellular contexts. Nature reviews Molecular cell biology.

[7] Wang KC, Chang HY (2011). Molecular mechanisms of long noncoding RNAs. Molecular cell.

[8] Rafiee A, Riazi-Rad F, Havaskary M, Nuri F (2018). Long noncoding RNAs: regulation, function and cancer. Biotechnology & genetic engineering reviews.

[9] Evans JR, Feng FY, Chinnaiyan AM (2016). The bright side of dark matter: lncRNAs in cancer. The Journal of clinical investigation.

[10] Schmitt AM, Chang HY (2016). Long Noncoding RNAs in Cancer Pathways. Cancer cell.

[11] Zhao R, Li FQ, Tian LL, Shang DS, Guo Y, Zhang JR (2019). Comprehensive analysis of the whole coding and non-coding RNA transcriptome expression profiles and construction of the circRNA-lncRNA co-regulated ceRNA network in laryngeal squamous cell carcinoma. Functional & integrative genomics.

[12] Chen J, Shen Z, Deng H, Zhou W, Liao Q, Mu Y (2018). Long non-coding RNA biomarker for human laryngeal squamous cell carcinoma prognosis. Gene.

[13] Xu J, Guo J, Jiang Y, Liu Y, Liao K, Fu Z (2019). Improved characterization of the relationship between long intergenic non-coding RNA Linc00152 and the occurrence and development of malignancies. Cancer medicine.

[14] Nishizawa Y, Konno M, Asai A, Koseki J, Kawamoto K, Miyoshi N (2018). Hypoxia stimulates the cytoplasmic localization of oncogenic long noncoding RNA LINC00152 in colorectal cancer. International journal of oncology.

[15] Huang Y, Luo H, Li F, Yang Y, Ou G, Ye X (2018). LINC00152 down-regulated miR-193a-3p to enhance MCL1 expression and promote gastric cancer cells proliferation. Bioscience reports.

[16] Wu J, Shuang Z, Zhao J, Tang H, Liu P, Zhang L (2018). Linc00152 promotes tumorigenesis by regulating DNMTs in triple-negative breast cancer. Biomedicine & pharmacotherapy = Biomedecine & pharmacotherapie.

[17] Zhu Z, Dai J, Liao Y, Ma J, Zhou W (2018). Knockdown of Long Noncoding RNA LINC00152 Suppresses Cellular Proliferation and Invasion in Glioma Cells by Regulating miR-4775. Oncology research.

[18] Feng S, Zhang J, Su W, Bai S, Xiao L, Chen X (2017). Overexpression of LINC00152 correlates with poor patient survival and knockdown impairs cell proliferation in lung cancer. Scientific reports.

[19] Deng X, Zhao XF, Liang XQ, Chen R, Pan YF, Liang J (2017). Linc00152 promotes cancer progression in hepatitis B virus-associated hepatocellular carcinoma. Biomedicine & pharmacotherapy = Biomedecine & pharmacotherapie.

[20] Wu Y, Tan C, Weng WW, Deng Y, Zhang QY, Yang XQ (2016). Long non-coding RNA Linc00152 is a positive prognostic factor for and demonstrates malignant biological behavior in clear cell renal cell carcinoma. American journal of cancer research.

[21] Zhao L, Chi WW, Cao H, Meng WX, Cui WN, Wang BS (2019). [Expression of long-chain non-coding RNA LINC00152 in laryngeal squamous cell carcinoma and its clinical significance]. Lin chuang er bi yan hou tou jing wai ke za zhi = Journal of clinical otorhinolaryngology, head, and neck surgery.

[22] Wang JX, Yang Y, Li K (2018). Long noncoding RNA DANCR aggravates retinoblastoma through miR-34c and miR-613 by targeting MMP-9. Journal of cellular physiology.

[23] Mei J, Xu R, Hao L, Zhang Y (2019). MicroRNA-613: A novel tumor suppressor in human cancers. Biomedicine & pharmacotherapy = Biomedecine & pharmacotherapie.

[24] Nadal A, Cardesa A (2003). Molecular biology of laryngeal squamous cell carcinoma. Virchows Archiv : an international journal of pathology.

[25] Lyu K, Xu Y, Yue H, Li Y, Zhao J, Chen L (2019). Long Noncoding RNA GAS5 Acts As A Tumor Suppressor In Laryngeal Squamous Cell Carcinoma Via miR-21. Cancer management and research.

[26] Li J, Sun S, Chen W, Yuan K (2019). Small Nucleolar RNA Host Gene 12 (SNHG12) Promotes Proliferation and Invasion of Laryngeal Cancer Cells via Sponging miR-129-5p and Potentiating WW Domain-Containing E3 Ubiquitin Protein Ligase 1 (WWP1) Expression. Medical science monitor : international medical journal of experimental and clinical research.

[27] Huang Y, Zhang C, Zhou Y (2019). LncRNA MIR100HG promotes cancer cell proliferation, migration and invasion in laryngeal squamous cell carcinoma through the downregulation of miR-204-5p. OncoTargets and therapy.

[28] Tay Y, Rinn J, Pandolfi PP (2014). The multilayered complexity of ceRNA crosstalk and competition. Nature.

[29] Zhang YH, Fu J, Zhang ZJ, Ge CC, Yi Y (2016). LncRNA-LINC00152 down-regulated by miR-376c-3p restricts viability and promotes apoptosis of colorectal cancer cells. American journal of translational research.

[30] Sun Z, Guo X, Zang M, Wang P, Xue S, Chen G (2019). Long non-coding RNA LINC00152 promotes cell growth and invasion of papillary thyroid carcinoma by regulating the miR-497/BDNF axis. Journal of cellular physiology.

[31] Sun K, Hu P, Xu F (2018). LINC00152/miR-139-5p regulates gastric cancer cell aerobic glycolysis by targeting PRKAA1. Biomedicine & pharmacotherapy = Biomedecine & pharmacotherapie.

[32] Liu X, Yidayitula Y, Zhao H, Luo Y, Ma X, Xu M (2018). LncRNA LINC00152 promoted glioblastoma progression through targeting the miR-107 expression. Environmental science and pollution research international.

[33] Liu D, Gao M, Wu K, Zhu D, Yang Y, Zhao S (2019). LINC00152 facilitates tumorigenesis in esophageal squamous cell carcinoma via miR-153-3p/FYN axis. Biomedicine & pharmacotherapy = Biomedecine & pharmacotherapie.

[34] Chen P, Fang X, Xia B, Zhao Y, Li Q, Wu X (2018). Long noncoding RNA LINC00152 promotes cell proliferation through competitively binding endogenous miR-125b with MCL-1 by regulating mitochondrial apoptosis pathways in ovarian cancer. Cancer medicine.

[35] Sang Q, Liu X, Sun D (2018). Role of miR-613 as a tumor suppressor in glioma cells by targeting SOX9. OncoTargets and therapy.

[36] Liu H, Chen K, Wang L, Zeng X, Huang Z, Li M (2019). miR-613 inhibits Warburg effect in gastric cancer by targeting PFKFB2. Biochemical and biophysical research communications.

[37] Jiang X, Wu J, Zhang Y, Wang S, Yu X, Li R (2018). MiR-613 functions as tumor suppressor in hepatocellular carcinoma by targeting YWHAZ. Gene.

[38] Xiong H, Yan T, Zhang W, Shi F, Jiang X, Wang X (2018). miR-613 inhibits cell migration and invasion by downregulating Daam1 in triple-negative breast cancer. Cellular signalling.

[39] Wang J, Yang S, Ge W, Wang Y, Han C, Li M (2018). MiR-613 suppressed the laryngeal squamous cell carcinoma progression through regulating PDK1. Journal of cellular biochemistry.

